# Histological analysis of induced membranes in patients whose bone defects were treated with the Masquelet technique to identify factors affecting the vascularity of induced membranes

**DOI:** 10.1186/s13018-021-02404-7

**Published:** 2021-04-13

**Authors:** Takahiro Niikura, Naoe Jimbo, Masato Komatsu, Keisuke Oe, Tomoaki Fukui, Tomoyuki Matsumoto, Shinya Hayashi, Takehiko Matsushita, Yoshitada Sakai, Tomoo Itoh, Ryosuke Kuroda

**Affiliations:** 1 Department of Orthopaedic Surgery, Kobe University Graduate School of Medicine, 7-5-1 Kusunoki-cho, Chuo-ku, Kobe, 650-0017 Japan; 2 Department of Diagnostic Pathology, Kobe University Graduate School of Medicine, 7-5-1 Kusunoki-cho, Chuo-ku, Kobe, 650-0017 Japan; 3 Division of Rehabilitation Medicine, Kobe University Graduate School of Medicine, 7-5-1 Kusunoki-cho, Chuo-ku, Kobe, 650-0017 Japan

**Keywords:** Masquelet technique, Induced membrane, Bone defect, Vascularity, Histology

## Abstract

**Background:**

Rich vascularity of the induced membrane (IM) is important for Masquelet reconstruction surgery. The factors affecting IM vascularity are not completely understood. This study aimed to investigate these factors using histological samples of human IMs.

**Methods:**

We retrospectively evaluated 36 patients whose bone defects were treated using the Masquelet technique. Two clinical pathologists analyzed histological sections of IM pieces (1 cm^2^). The number of blood vessels per 1 mm^2^ was counted and compared among men and women, femur or tibia, with and without free flap surgery, antibiotic impregnation to the cement, osteogenesis inside the membrane, smoking, and diabetes mellitus. The number of blood vessels within the same patient was compared among different time points. Correlation analysis was performed among blood vessel numbers and patient age, duration of cement spacer placement, and histological grading scales (inflammation, foreign body reaction, and fibrosis).

**Results:**

IM formation with rich vascularity and some inflammation, foreign body reaction, and fibrosis were histologically confirmed in all patients. We found 37.4 ± 19.1 blood vessels per 1 mm^2^. The number of blood vessels was significantly lower in patients with than in those without free flap surgery; it was higher in patients with osteogenesis inside the IM. No significant correlations were found in any of the analyses.

**Conclusion:**

Sex, patient age, smoking, diabetes mellitus, femur or tibia, duration of cement spacer placement, and antibiotic impregnation to the cement did not affect IM vascularization. IM vascularization was reduced in patients with than in those without free flap surgery.

## Background

Reconstruction of a large bone defect caused by trauma, infection, or tumor is one of the most challenging problems in orthopedic surgery. Bone lengthening using the Ilizarov method (distraction osteogenesis and callotasis) [1, 2] and vascularized fibula grafts [3, 4] are limited treatment options. An option to reconstruct large bone defects was proposed by Alain-Charles Masquelet [5–7]. A biological membrane forms around a cement spacer that is placed in a large bone defect. This membrane is called an induced membrane (IM). Once the IM has formed, the cement is removed and bone grafting is performed. The IM serves as a conduit for cells and provides a favorable environment for bone graft osseointegration. IM formation around a cement spacer is the key component of Masquelet reconstruction surgery to treat bone defects [5–8]. Animal studies have shown that the IM possesses estrogenic and osteoinductive activities and a rich vascularity [9–13].

Information concerning the properties of the IM in a clinical setting is limited. In particular, analyses of histological samples of human IM specimens are limited [14–16]. Therefore, we sought to analyze human IM samples histologically. Rich vascularity is an important component of the IM for successful bone reconstruction. The diamond concept of bone regeneration considers vascularity to be an essential component [17, 18]. However, the factors affecting the vascularity of the IM are not fully understood. Hence, this study aimed to investigate the factors that affect the vascularity of the IM using histological samples of human IM.

## Methods

### Patient inclusion

This retrospective study involving human participants was conducted in accordance with the ethical standards of the institutional and national research committee and with the 1964 Helsinki Declaration and its later amendments or comparable ethical standards. The Ethics Committee of Kobe University approved this study (approval number: B190237). The requirement for informed consent was waived due to the retrospective nature of the study. We included all patients whose bone defects were treated using the Masquelet technique at our department between August 2016 and October 2020.

### Histological specimens

During the second surgery (removal of the cement and bone grafting), 1-cm^2^ pieces of the IM that were in contact with the cement spacer were harvested and immersed in 10% neutral buffered formalin. Histological sections were stained with hematoxylin and eosin and analyzed by two clinical pathologists.

### Blood vessel count

The clinical pathologists counted the number of blood vessels per 1 mm^2^ within the IM at the locations at which the capillary density was the highest. A section with the highest capillary density was selected to analyze patients with two or more samples.

### Histological analyses

Histological findings of inflammation, foreign body reaction, and fibrosis were assessed using a semi-quantified grading scale of zero, one, two, and three. Grade three indicated the highest findings of inflammation, foreign body reaction, and fibrosis, and zero indicated no findings.

### Analyzed variables

The patients’ sex, age, morbidity accounting for the bone defect, free flap application to the affected limb, affected site (bone), impregnation of antibiotics to the cement spacer, duration of cement placement, smoking habit, and comorbidity of diabetes mellitus (DM) or peripheral artery disease (PAD) were investigated using the medical charts. Osteogenesis inside the IM, the inflammation grade, foreign body reaction, and fibrosis were investigated using the histological analysis reports of the IM samples.

### Clinical course

Bony union in the involved patients was assessed radiologically and clinically. The time point of bony union assessment was set at 6 months after the second stage of surgery (autologous bone grafting). Radiological bony union was corticalization of the grafted bone, and no gap between the grafted and original bone, which was observed in three or four cortices using orthogonal (antero-posterior and medio-lateral) radiographs. Clinical bony union was no pain with full weight-bearing for those with lower extremity fractures, and no pain with full activities for those with upper extremity fractures. Bony union was defined as achieving both radiological and clinical bony union. Three experienced orthopedic trauma surgeons individually assessed the bony union. If two or three surgeons assessed a bony union, that sample was defined as a bony union. Patients with less than a 6-month period after the second stage surgery were excluded from this assessment.

### Statistical analyses

Data are shown as mean ± standard deviation. IBM SPSS Statistics Software Ver. 26J (IBM Japan, Tokyo, Japan) was used for the statistical analyses. The number of blood vessels was compared between men and women, with and without free flap surgery, with and without antibiotic impregnation to the cement, with and without osteogenesis inside the IM, with and without smoking, with and without DM, and based on the affected site (femur or tibia) using the Mann–Whitney *U* test. Correlation analysis was performed among the number of blood vessels and clinical information, including patient age, duration of cement placement, grading scale of inflammation, foreign body reaction, and fibrosis, using Spearman’s rank correlation coefficient. These analyses were performed separately when the earlier time points for the five patients with cement replacements were adopted and when the later time points for the five patients with cement replacements were adopted. The number of blood vessels within the same patient was compared among the different time points using the Wilcoxon signed-rank test. A *p* value < 0.05 was considered statistically significant (two-sided test).

## Results

### Patient characteristics

Thirty-six patients (27 male, 9 female; age, 52.1 ± 17.5 years) were included (Table 1). The morbidities accounting for the bone defects were infected nonunion (16 patients), osteomyelitis (8 patients), uninfected nonunion (4 patients), comminuted open fracture (3 patients), septic arthritis (3 patients), and infected delayed union (2 patients). Five samples were repeatedly harvested from the same patients because the cement spacers were replaced with new ones at the first stage. The cement spacers were replaced because of conversion of external to internal fixation in one, biopsy to exclude potential infection in one, and additional free flap surgeries for three patients. Information for the earlier time points for the five patients with cement replacements is shown in Table 1.

**Table 1:** Patients’ characteristics and data of histological analyses

Case	Sex	Age	Affected site	Duration of the cement placement (days)	Free flap	Antibiotics within the cement	Smoking	DM	Osteogenesis within the membrane	Inflammation	Foreign body reaction	Fibrosis	Blood vessel counts per 1mm^**2**^
**1**	M	43	Tibia	83	+	+	+		+	1	1	2	55
**2**	M	22	Ilium	74		+				1	1	2	30
**3**	M	58	Femur	46			+			1	2	1	70
**4**	M	70	Tibia	116		+	+	+	+	2	1	2	45
**5**	F	66	Calcaneus	49		+				2	1	1	15
**6**	M	32	Tibia	49		+	+			1	2	1	20
**7**	M	48	Tibia	67	+	+	+			2	2	1	35
**8**	M	23	Femur	46			+			2	2	1	30
**9**	F	73	Tibia	47		+	+			2	1	2	55
**10**	F	61	Tibia	53		+	+			2	2	1	25
**11**	M	55	Tibia	61		+	+			2	0	1	60
**12**	M	65	Tibia	50		+	+			1	1	2	30
**13**	M	55	Tibia	56		+	+			1	1	2	15
**14**	M	47	Knee	98		+	+	+		2	1	1	55
**15**	M	51	Femur	54		+	+			1	3	2	20
**16**	M	17	Tibia	63		+				2	0	2	20
**17**	M	52	Tibia	126	+	+				2	2	1	15
**18**	F	72	Femur	56		+				2	1	1	30
**19**	M	54	Knee	37	+	+	+			2	1	2	30
**20**	M	14	Clavicle	84		+				2	3	2	50
**21**	M	42	Tibia	100	+	+				2	3	1	20
**22**	M	60	Tibia	72		+	+		+	1	1	2	55
**23**	M	31	Fibula	63			+			1	1	2	45
**24**	M	44	Tibia	56		+	+		+	2	1	2	92
**25**	M	54	Tibia	112		+	+			1	2	2	38
**26**	M	69	Tibia	65	+	+	+	+		1	1	2	17
**27**	M	63	Tibia	90		+	+			2	1	3	55
**28**	F	71	Tibia	83	+	+				1	1	1	10
**29**	F	64	Tibia	70		+				1	1	2	28
**30**	F	45	Femur	37		+				2	1	2	56
**31**	M	58	Femur	57		+	+	+		2	1	3	53
**32**	M	25	Femur	110			+			1	0	3	22
**33**	F	67	Tibia	56		+				3	3	2	40
**34**	M	61	Calcaneus	84	+	+	+	+		1	1	3	50
**35**	M	53	Tibia	125	+	+	+			2	2	1	9
**36**	F	90	Femur	63		+		+		2	1	2	53

*M* male, *F* female, *Knee* knee joint, *DM* diabetes mellitus. Grading scale of the inflammation, foreign body reaction, and fibrosis is shown as 0 to 3. Cement placement was done twice for cases 1, 13, 15, 17, and 19. Data of the 1st placement is shown

The affected sites were the tibia (21 patients), femur (8 patients), knee joint (2 patients), calcaneus (2 patients), and pubis, clavicle, and fibula (1 patient each). The cement placement duration, defined as the number of days from the first-stage surgery in which the cement spacer was placed, to the second stage surgery in which the cement spacer was removed and bone grafting was done, was 71.1 ± 24.7 days. Nine patients (25.0%) underwent free flap surgery. Thirty-two patients (88.9%) received antibiotic impregnation. Twenty-four patients (66.7%) had a smoking habit. Six patients (16.7%) had a DM comorbidity. None of the patients had a PAD comorbidity.

### Clinical course

Four patients with a time period of less than 6 months after the second-stage surgery were excluded from the assessment of bony union. Thirty of 32 patients (93.8%) achieved bony union. The two patients who did not achieve bony union included one who needed an additional Masquelet reconstruction surgery to obtain bony union, and one who experienced a postoperative infection 1 month after the second surgery.

### Histological findings

IM formation was confirmed histologically in all patients. A two-layer structure was noted in all patients. Synovial-like structure at the surface that was in contact with the cement was identified in 19 patients (52.8%). Fibrin deposition was shown in 28 patients (77.8%).

### Histological data

**Fig. 1:**
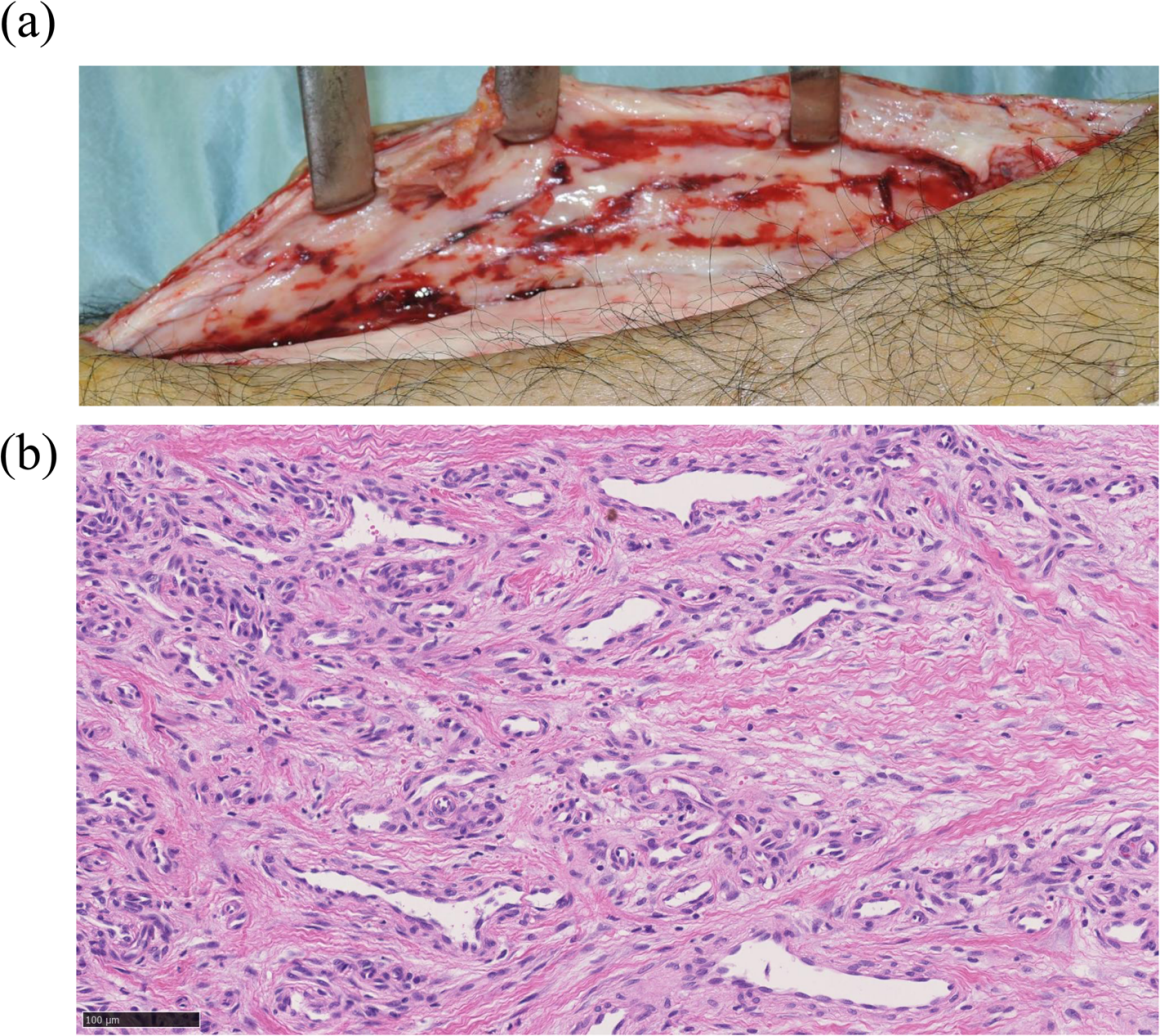
Representative images of rich vascularity of the induced membrane. **a** The image shows the induced membrane with rich vascularity. **b** The image shows histological findings of rich blood vessel formation (hematoxylin and eosin staining). The scale bar represents 100 μm

**Fig. 2:**
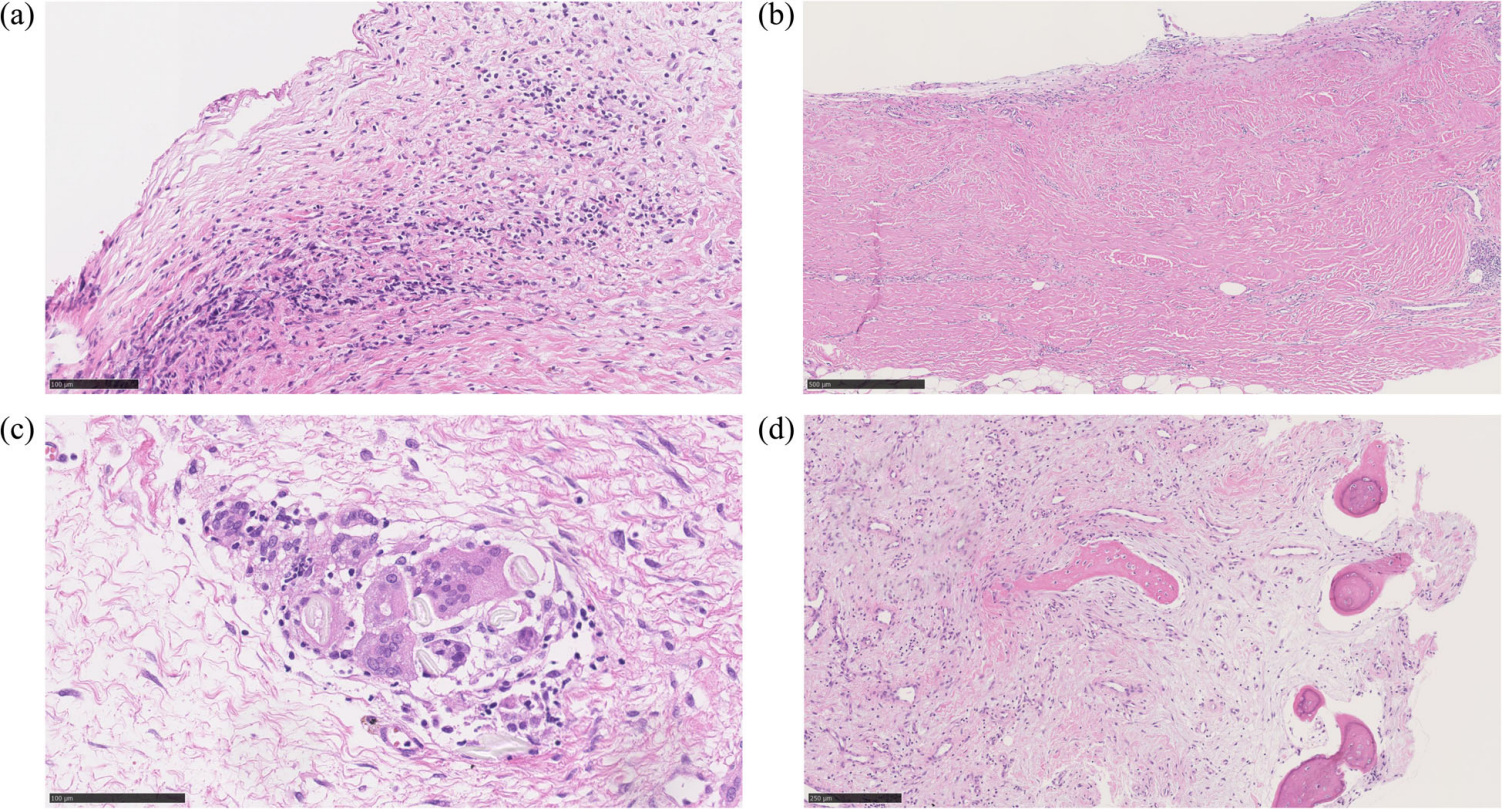
Representative histological images of the induced membranes. **a** Inflammation (HE), **b** fibrosis (HE), **c** foreign body reaction (HE), and **d** osteogenesis inside the induced membrane (HE). HE, hematoxylin and eosin staining. The scale bars represent **a** 100 μm, **b** 500 μm, **c** 100 μm, and **d** 250 μm

Blood vessel formation was noted in all patients (Fig. 1). The number of blood vessels per 1 mm^2^ was 37.4 ± 19.1. Inflammation and fibrosis were observed in all patients, and foreign body reaction was found in 33 patients (91.7%) (Fig. 2). The histological grading of inflammation, fibrosis, and foreign body reaction are shown in Table 1. Osteogenesis inside the IM was noted in four patients (11.1%) (Fig. 2).

### Comparison analyses

No significant differences were found in the number of blood vessels among men and women, with and without antibiotic impregnation in the cement, with and without smoking, with and without DM, and the femur or tibia (Table 2). There were no significant differences in the number of blood vessels within the same patient at different time points. On the other hand, there were significant differences in the number of blood vessels in patients with and without free flap surgery and in those with and without osteogenesis inside the IM. Patients without free flap surgery had a larger number of blood vessels than those with free flap surgery did (*p* = 0.043). Patients with osteogenesis inside the IM had a larger number of blood vessels than those without osteogenesis inside the IM did (*p* = 0.021).

**Table 2:** Comparisons of blood vessel counts per 1 mm^2^

	*n*	Mean	S.D.
(a) Sex			
Male	27	38.4	20.0
Female	9	34.7	17.3
(b) Free flap			
Free flap (−)	27	41.0	18.8
Free flap (+)	9	26.8	16.9
(c) Antibiotics within the cement			
Antibiotics (−)	4	41.8	21.1
Antibiotics (+)	32	36.9	19.2
(d) Osteogenesis inside the membrane			
Osteogenesis (−)	32	34.4	16.9
Osteogenesis (+)	4	61.8	20.7
(e) Smoking			
Smoking (−)	12	30.6	15.8
Smoking (+)	24	40.9	20.0
(f) Diabetes mellitus			
DM (−)	30	35.8	19.8
DM (+)	6	45.5	14.4
(g) Tibia vs. femur			
Tibia	21	35.2	21.1
Femur	8	41.8	18.5
(h) Patients with cement placement twice			
First	5	31.0	19.8
Second	5	24.6	7.5

### Correlation analyses

**Fig. 3:**
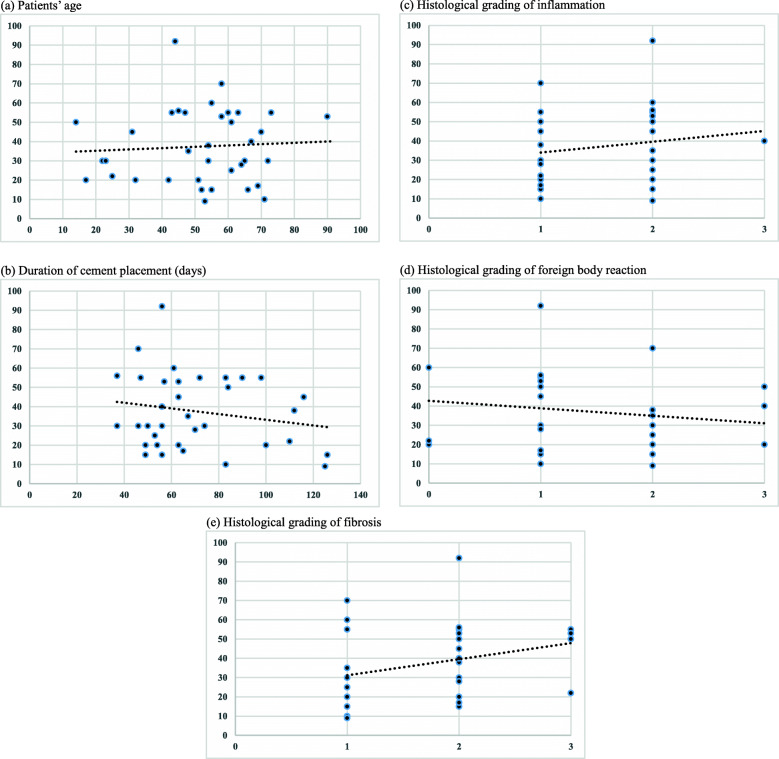
Correlations among blood vessel counts per 1 mm^2^ of induced membranes and the patients’ characteristics. **a** Patients’ age (*x* axis) and blood vessel counts (*y* axis), correlation coefficient = 0.017, *p* = 0.920; **b** duration of cement placement (days) (*x* axis) and blood vessel counts (*y* axis), correlation coefficient = − 0.141, *p* = 0.412; **c** histological grading of inflammation (*x* axis) and blood vessel counts (*y* axis), correlation coefficient = 0.166, *p* = 0.332; **d** histological grading of foreign body reaction (*x* axis) and blood vessel counts (*y* axis), correlation coefficient = −0.199, *p* = 0.246; and **e** histological grading of fibrosis (*x* axis) and blood vessel counts (*y* axis), correlation coefficient = 0.292, *p* = 0.084

No significant correlations were found in any of the analyses (Fig 3). The correlation coefficient between blood vessel counts per 1 mm^2^ and other variables were patients’ age, 0.017 (*p* = 0.920); duration of cement placement, − 0.141 (*p* = 0.412); histological grading of inflammation, 0.166 (*p* = 0.332); histological grading of foreign body reaction, − 0.199 (*p* = 0.246); and histological grading of fibrosis, 0.292 (*p* = 0.084).

## Discussion

This study confirmed that IM possessed rich vascularity and revealed that the vascularity of the IM was not influenced by patient factors, including sex, age, smoking, and DM, in the human samples. There were no significant differences in the IM blood vessel counts between the femur and tibia sites, and the first and second placements of cement in the same patients. These results suggest that the Masquelet technique can be applied clinically to a wide range of patients because the vascularity of the IM was not associated with these patient factors.

Aho et al. have reported that the vascularity of the IM decreases when the duration of the cement spacer placement increases (*n*=14) [14]. Their findings differ from our results. This contradiction may be explained by the methodology used to assess vascularity. Aho et al. quantified the proportional vascularity area in comparison to the membrane stroma. In contrast, we counted the number of blood vessels. We found no significant correlation between vascularity and the duration of cement placement. Recently, Gindraux et al. have reported that bone healing is not disturbed when the second surgical stage of Masquelet’s technique is performed at a later time point [19]. The median waiting period between the first and second surgical stage ranged from 5.8 to 14.7 months. This finding was reported in their limited case series of 34 patients. However, this finding suggests that the length of time that cement spacers remain in the treatment site does not influence the activities of the IM. Our results demonstrated that the length of time that the cement spacers remained in the treatment site did not affect the number of blood vessels in the IM. This finding suggests that a longer waiting period before the second surgery does not disturb the vascularity of the IM. Further, it may suggest that a longer waiting period before the second surgery does not disturb bone healing.

Impregnation of the cement with antibiotics is recognized as an effective method of local antibiotic delivery to treat the infection of bone and joint [20]. The Masquelet technique is often applied to treat osteomyelitis [21] or infected nonunion; therefore, impregnation of antibiotics in the cement spacer is usually performed by the surgeon during the first stage of the Masquelet reconstruction surgery, which is similar to our method. Nau et al. have demonstrated that the thickness and proportion of elastic fibers in the IM are influenced by the type of cement and supplemental antibiotics administered in a study using a critical-size defect model of the rat femur [22]. Their results can be interpreted to indicate that the addition of antibiotics to the cement does not impair the vascularity of IM. Recently, Roukoz et al. have shown that the addition of antibiotics to the cement was effective in controlling infection, and did not alter the membrane’s activity in a rat infected nonunion model [23]. The results of our study revealed that the addition of antibiotics to the cement did not impair the vascularity of the IM in human clinical samples. This finding can be informative for surgeons.

We found a significant difference in the blood vessel count between patients who received and did not receive free flap surgery. To our knowledge, our study is the first to report this finding. However, we found no differences in clinical results such as bony union between the patients with and without free flap surgeries. Therefore, it is unclear whether a reduced number of blood vessels in patients with free flap surgeries affect bone formation. This result may be interpreted as follows: a patient requiring free flap surgery inherently has a worse soft tissue condition and worse vascularity compared to a patient who does not require free flap surgery. Free flap surgery is undertaken to treat the damaged soft tissue; however, the inherently reduced vascularity and vasculogenesis potential of the soft tissue may result in a reduced number of blood vessels.

Aho et al. have mentioned that endochondral ossification is found in the IM histologically [14]. Gruber et al. have reported histological findings which show trabecular bone in 33.3% of the IM specimens [24]. We found similar results in limited samples. A significantly higher number of blood vessels were detected in the IM samples with osteogenesis inside the membrane. This can be explained with a cause-and-effect mechanism. Rich vascularity may induce bone formation inside the IM. Rich vascularity and osteogenic and osteoinductive activity [10, 11, 14, 15, 19, 24] are key components of IM. Although the relationship and balance between vascularity and osteogenic/osteoinductive activity is not clear, our findings may provide clues to obtain a better understanding of this relationship.

Aho et al. have described the IM as a foreign body granulation tissue membrane, foreign body-induced granulation tissue membrane, and an induced granulation tissue membrane [14]. They postulate that the younger membrane contains granulation tissue and the older membrane contains a more uniform fibrous tissue. Pelissier et al. have reported that acute inflammation and foreign body reaction are dominant in younger membranes [9]. Recently, Vitiello et al. observed foreign body reaction and IM formation following silver-coated knee megaprosthesis reconstruction [25]. We also found the presence of inflammation, foreign body reaction, and fibrosis in the IM samples histologically. Consequently, we employed a grading scale to evaluate them. These findings may reflect the maturity of the IM in combination. We found no significant correlation between the grading scales of inflammation, foreign body reaction, fibrosis, and blood vessel numbers. Our results suggested that blood vessel formation had occurred irrespective of the maturity of the IM, similar to the duration of cement spacer placement.

This study’s strength is the relatively large number of human IM samples (*n* = 36). For example, Cuthbert et al. [15] examined 8 patients, Gruber et al. [24] included 12 patients, and Aho et al. [14] included 14 patients. In addition, we included IM data from repeated harvest of samples from the same patients. The limitation of this study is that we focused on the vascularity of the IM. We did not perform a comparative study between responders and non-responders [26]. This was a retrospective study that was conducted at one institution. Biases, such as the selection bias and the generalizability bias, may have affected the results.

## Conclusions

In conclusion, IM vascularization was not affected by sex, patient age, smoking, DM, affected site of the bone defect, duration of cement spacer placement, and antibiotic impregnation to the cement. IM vascularization was reduced in patients who received compared with those who did not receive free flap surgery. Our results suggest a relationship between vascularity and osteogenesis inside the IM and provide a basis for further studies in this.

## Data Availability

The data used and/or analyzed during the current study are available from the corresponding author on reasonable request.

## Abbreviations

DM: Diabetes mellitus
IM: Induced membrane
PAD: Peripheral artery disease
